# Resolving Multi-Asperity Contacts at the Nanoscale through Super-Resolution Fluorescence Imaging

**DOI:** 10.1021/acs.jpclett.3c02799

**Published:** 2024-02-12

**Authors:** Begüm Demirkurt, Dina Petrova, Dharmendar Kumar Sharma, Martin Vacha, Bart Weber, Daniel Bonn, Albert M. Brouwer

**Affiliations:** † van’t Hoff Institute for Molecular Sciences, University of Amsterdam, P.O. Box 94157, 1090 GD Amsterdam, The Netherlands; ‡ Tokyo Institute of Technology, Ookayama 2-12-1-S8, Meguro-ku, Tokyo 152-8552, Japan; § Advanced Research Center for Nanolithography (ARCNL), Science Park 106, 1098 XG Amsterdam, The Netherlands; ∥ Institute of Physics, University of Amsterdam, P.O. Box 94485, 1090 GL Amsterdam, The Netherlands

## Abstract

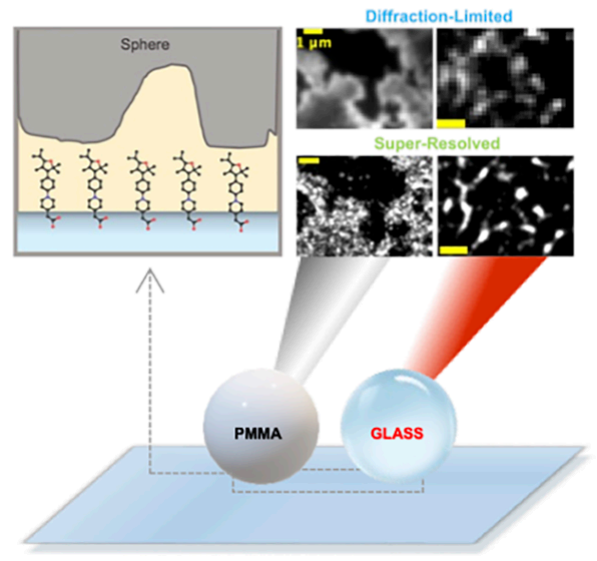

Contact mechanics, spanning nanometer to tectonic scales, faces long-standing challenges arising from multiscale random roughness, which hinders experimental validation of theories. Understanding multi-asperity rough contacts is vital for addressing catastrophic consequences of these contacts failing such as earthquakes and for diverse technological applications. To visualize such contacts, we introduce a super-resolution microscopy method utilizing spontaneous millisecond ON/OFF fluorescence blinking of contact-sensitive molecular rotor molecules immobilized on a glass coverslip. This technique achieves ∼55 nm lateral imaging resolution for rough poly(methyl methacrylate) and glass spheres on glass contacts. For soft polymer spheres due to large plastic deformation, the resolution improvement does not significantly affect the area of real contact. However, for hard glass spheres, the real contact area is found to be 2.4 times smaller than that found by diffraction-limited imaging. This study highlights, through direct visualization, the impact of material stiffness on the nanoscale structure within the area of real contact.

Friction is a ubiquitous phenomenon with many important consequences for human life and is responsible for an estimated 23% of the world’s energy consumption.^1^ Friction plays a key role in technological applications such as car tire–road contact, contacts of artificial hip joints used in orthopedics, nano- and micro-contacts in micro-electromechanical systems (MEMS) used in smartphones, navigation systems, biomedical devices, etc.^2^ Here, the challenge is to design interfaces for optimal friction and minimal wear. On much larger scales, contact mechanics also control geophysical phenomena such as earthquakes and landslides.^3,4^ In this context, the challenge is to predict catastrophic slip events that may endanger human lives and infrastructure. In all cases, the understanding of the contact mechanics is a central issue. The real area of contact formed during the mechanical contact of two surfaces is at the heart of understanding friction and the onset of sliding.^5^ The complexity of contact mechanics is that all surfaces, even mirror polished ones, possess roughness that is generally random and spans a wide range of length scales. The apparent contact area at a macroscopic scale and the real contact area at much smaller scales can therefore be very different.^6−8^ Because the real contact area determines the frictional resistance, this is a key quantity that needs to be understood.^3,9,10^

The earliest contact mechanics theories that are used to predict important parameters such as the real contact area, contact stress distribution, etc., either assume perfectly smooth contact interfaces by excluding roughness^11^ or include roughness in an idealized manner.^12,13^ Assumptions about the roughness can lead to incorrect estimations of the real contact area and, therefore, of the friction.^2,14^ Because of recent developments in these theoretical approaches, multiscale roughness can now be included in the models, for instance, by basing contact calculations on multiscale topography measurements, and this enables us to theoretically predict what length scales are critical for the contact structure.^8,15−20^ For instance, both boundary element model (BEM) calculations and the analytical approach developed by Persson predict that as the roughness surface slopes increase, contact patches become smaller and subjected to higher pressures. A natural limit of the local contact pressure is given by the hardness of the contacting materials. If the local pressure exceeds the hardness, the material deforms plastically.^8,19,20^ However, some of the assumptions of these recent models, such as idealized elasticity and/or plasticity, might not hold in reality.^3^ Therefore, in the absence of experimental methods that can achieve *in situ* imaging of multi-asperity rough contact interfaces at different length scales (at different resolutions), it is difficult to assess the accuracy of contact mechanics models.^17^ Furthermore, visualizing the area of real contact is a major experimental challenge; in particular, methods through which multiscale contacts can be visualized at the nanoscale are lacking.^21^

We recently developed a method for quantitatively imaging the real contact area using fluorescence microscopy, making use of the fluorescence enhancement (due to the suppression of nonradiative decay) of molecular rotors when they are confined in a contact.^3,9,10,23,38^ This allowed for a detailed comparison between different contact mechanics models.^3^ In a study of contacts between roughened polystyrene (PS) spheres and glass surfaces functionalized with surface-bound molecular rotors of the dicyanomethylenedihydrofuran (DCDHF) type, it was found that the contact patches were larger than the diffraction limit due to the plastic deformation of the asperities on the PS sphere.^3,23^ Therefore, the area of real contact can largely be resolved with regular microscopy for rough plastic contacts. However, for stiffer materials such as silica and silicon, both relevant for tribology-related challenges in the semiconductor industry, the hardness is more than an order of magnitude higher than for plastics, and this can lead to finer structure in the area of real contact that can no longer be resolved through regular, diffraction-limited, microscopy whose spatial resolution is ∼200–300 nm for visible light. The contact patches would be much smaller, and the diffraction limited imaging overestimates the real contact area.^2,22^ Observing such stiff contacts experimentally could place more stringent tests on the contact theories while at the same time opening up new opportunities to visualize the wear, adhesion, and slip of interfaces that play a key role in, i.e., computer chip production.

In this work, we extend the contact imaging technique described above to a subdiffraction limited resolution of ∼55 nm to study randomly rough multi-asperity contacts of PMMA and glass spheres on contact-sensitive glass. A surprisingly simple treatment of the initially dense DCDHF monolayer, namely, controlled photobleaching, leads to a suitably reduced label density of fluorophores, which blink by spontaneous switching between dark (OFF) and fluorescent (ON) states. Fluorescence blinking of dye molecules can have different causes. In our case, the most likely mechanism involves reversible transfer of electrons from and to trap states in the glass, which has been observed for other organic dyes.^24−28^ Details of our ongoing mechanistic studies will be reported elsewhere. The molecules that are in the ON state behave in the same way as the monolayer before bleaching and, most importantly, retain their contact sensitivity. Overall, the rigidochromic enhancement present at the single-molecule level and dynamics of blinking are found to be suitable for applying this system for super-resolution analysis based on single-molecule localization microscopy (SMLM), because of the probe’s high number of photons per blinking cycle and millisecond blinking dynamics.^29−33^

Monolayer samples of rigidochromic 2-(1-{4-[4-cyano-5-(dicyanomethylene)-2,2-dimethyl-2,5-dihydrofuran-3-yl]phenyl}piperidin-4-yl) acetic acid (DCDHF) coupled via amide bonds to aminopropylsilane-functionalized glass coverslips are prepared for regular fluorescence microscopy contact area imaging.^3,9,10^ A glass or polymer sphere, which is glued to the head of a rheometer, is pressed onto the functionalized glass coverslip by applying a controlled normal force while imaging the contact interface with an inverted epifluorescence wide-field microscope. We excite the molecules on the coverslip and detect emission from below to record the fluorescence images. In the absence of mechanical contact, the fluorescence is weak, but the intensity distribution is uniform (Figure 1A), as expected for a monolayer of dye molecules with a density of ∼0.08 nm^–2^.^3^ For super-resolution imaging, however, a lower coverage is needed to have approximately one molecule per diffraction-limited spot, which can be achieved by exposing the monolayer to intense continuous wave (CW) laser light at 488 nm (1 kW cm^–2^). Partial bleaching of the fluorescence leads to an almost constant intensity after 1 h, in which we observe a stable number of fluorescent molecules per frame, with slower photobleaching. Single-molecule images at the glass–air interface are recorded with the same laser light (1 kW cm^–2^) with 50 ms/frame (Figure 1D). This simple approach is summarized in Figure 1.

**Figure 1:**
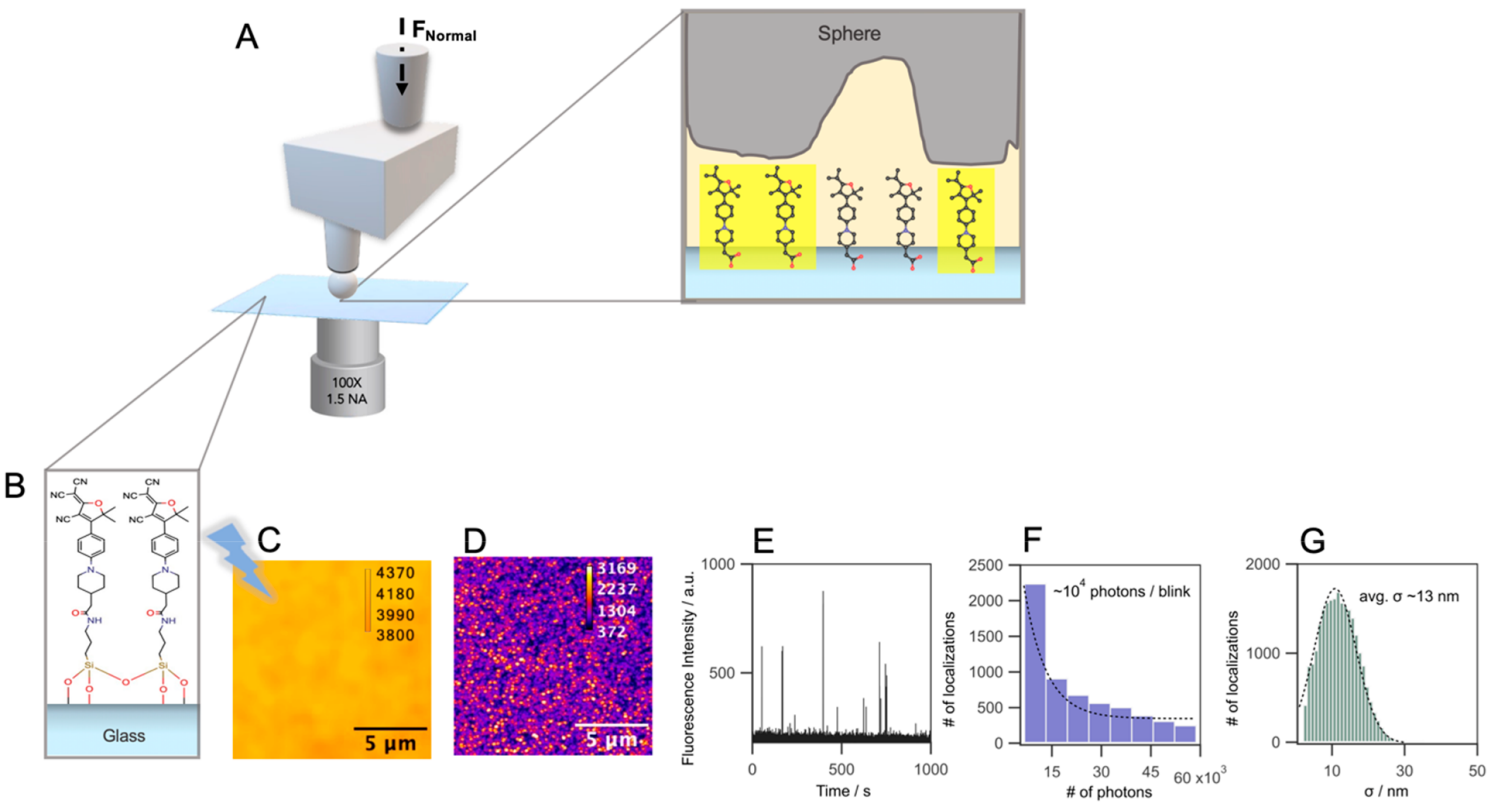
(A) Experimental setup for *insitu* mechanical contact visualization for a rough sphere on a glass contact via the diffraction-limited and super-resolution fluorescence imaging method described in this study. (B) Covalent attachment of DCDHF molecular rotors to an amino functional silane monolayer on a glass surface via amide coupling. (C) Fluorescence intensity image of a monolayer of DCDHF on the coverslip. (D) Maximum intensity-projected image of single-molecule blinking events in the bleached monolayer of DCDHF (no solvent added, 1 kW cm^–2^). (E) Fluorescence intensity time trace of a blinking single molecule in the bleached monolayer of DCDHF (no solvent added, 1 kW cm^–2^). (F) Histogram of the average number of photons per blinking cycle (ON time period). (G) Localization precision of blinking single molecules.

Laser irradiation leads to the irreversible bleaching of some of the molecules, which can be observed as a gradual decrease in intensity over time (Figure S1A). Most of the molecules, however, are bleached reversibly and remain mostly in long-lived dark state(s) from which they repopulate the fluorescent state. When single-molecule blinking events are projected in time by maximum intensity as shown in Figure 1D, a high degree of coverage of the entire area with blinking molecules is observed. Then, 20 000 images of the “blinking” monolayer under ambient conditions are collected for super-resolution analysis (ThunderSTORM plug-in in ImageJ)^34^ at the end of the second hour of bleaching. The average of the estimated localization precision for ∼10^4^ localizations is found to be ∼13 nm at the glass–air interface. The calculated average number of photons per blink (Figure 1F) is well in the range of ∼8000–12000 photons per event required for a good localization probe.^30^ In addition to the satisfactory photon numbers, the survival fraction (related to the active emitter concentration) and duty cycle (ratio between the number of molecules in the fluorescent ON state and OFF state) are the other important parameters that determine the final resolution in the super-resolved image and are observed to be satisfied for single DCDHF molecules in the partially photobleached monolayer (Figure S1).^30,33^ Detailed results and information about the single-molecule localization properties of the surface-bound DCDHF molecules are presented in the Supporting Information.

For super-resolution imaging of mechanical contacts, maintaining the rigidochromic sensitivity of molecules at the single-molecule level is key. To investigate this, we initially determined the basic fluorescence properties of individual molecules. The single-molecule emission spectra, depicted in Figure S2A, appear to be notably narrower than the bulk spectrum of the original monolayer before bleaching. The full width at half-maximum (fwhm) varies from molecule to molecule, and emission maxima of different molecules are also different with shifts of 8–10 nm, which is not uncommon for single molecules in a locally heterogeneous molecular environment.^35−37^ By summing the spectra of all of the molecules recorded, we obtained a spectrum that is indistinguishable from the spectrum of the original sample before bleaching.

The contact sensitivity of the DCDHF-based molecular rotor originates from its capability to undergo intramolecular rotations leading to nonradiative decay, thereby strengthening fluorescence when the rotors are confined.^38−41^ At the glass–air interface, molecular motion is partially restricted and fluorescence is readily detectable (Figure 1C). To enhance contrast and molecular motion, we introduced a low-viscosity solvent, dimethyl sulfoxide (DMSO), at the interface, simultaneously mitigating refractive index mismatches (*n*_D_^20^_glass_ = 1.52, *n*_D_^20^_PMMA_ = 1.49, and *n*_D_^20^_DMSO_ = 1.48). The presence of DMSO leads to a reduction in fluorescence intensity during the ON time, as expected for a molecular rotor (Figure S3). The fluorescence spectrum obtained by summing the spectra of single molecules is identical to that recorded for the original monolayer of DCDHF molecules immersed in DMSO (Figure S2B) and red-shifted from the spectrum in the “dry” state. When all are solvated by DMSO, the remaining single molecules in the partially bleached monolayer show less heterogeneity. In addition to these results, the mechanical contact sensitivity of single molecules is verified *in situ* during a contact visualization experiment evidenced by the observation of almost full recovery of molecules for two cycles of “bleaching–lifting the contact–restoring the same contact” experiments as described in Figure S4. Therefore, we conclude that the surface-bound rotor molecules that are detected after having been switched to the OFF state and returned to the ON state retain their contact sensitivity and that molecular fluorescence properties have not been altered. Eventually, the combination of the desired SMLM properties and preserved rigidochromic sensitivity at the single-molecule level allows us to use the “blinking monolayer” for super-resolution imaging of mechanical contacts.

After characterization of the probe, we apply our super-resolution approach to visualize the real contact area of rough PMMA and glass spheres on the functional glass coverslip as depicted in Figure 1A. We start with “conventional” imaging by means of the molecular rotor method.^9^ First, we create an interface by pressing a roughened PMMA sphere (0.5 mm in diameter) against the DCDHF-functionalized glass coverslip (before photobleaching and in the presence of DMSO) with a controlled normal force of ∼50 mN. At the contact points, surface-bound rotor molecules are confined by contacting with asperities on the surface of the sphere, which enhances the fluorescence via turning off the nonradiative decay channel(s). In this way, the diffraction-limited contact image of the PMMA sphere was recorded as shown in Figure 2A. After this reference measurement via conventional imaging, we bleach the monolayer in contact with PMMA by exposing it to intense CW laser light (488 nm, 1 kW cm^–2^). We then record a stack of ∼20 000 images of blinking rotor molecules under mechanical contact with 100 ms/frame (under irradiation at 488 nm, 1 kW cm^–2^), perform single-molecule localization, and reconstruct the image by using the locations of molecules. Although molecules in the noncontact regions also show blinking, those in contact are selected in the SMLM data processing based on their high brightness. The reconstructed super-resolved image is represented in Figure 2B. The obtained image closely resembles the contact area image obtained by regular fluorescence microscopy. To assess the resolution, Fourier ring correlation (FRC) analysis^42,43^ of the super-resolved image is performed from which an average resolution of 56 nm is found, based on a correlation value of 0.143 in the fixed threshold method as represented in Figure S5D. The molecular density is calculated as ∼1100 molecules/μm^2^ for the PMMA sphere-on-glass contact, which allows the theoretical resolution of ∼61 nm according to the Nyquist–Shannon theorem, which agrees well with the calculated resolution obtained by FRC analysis.

**Figure 2:**
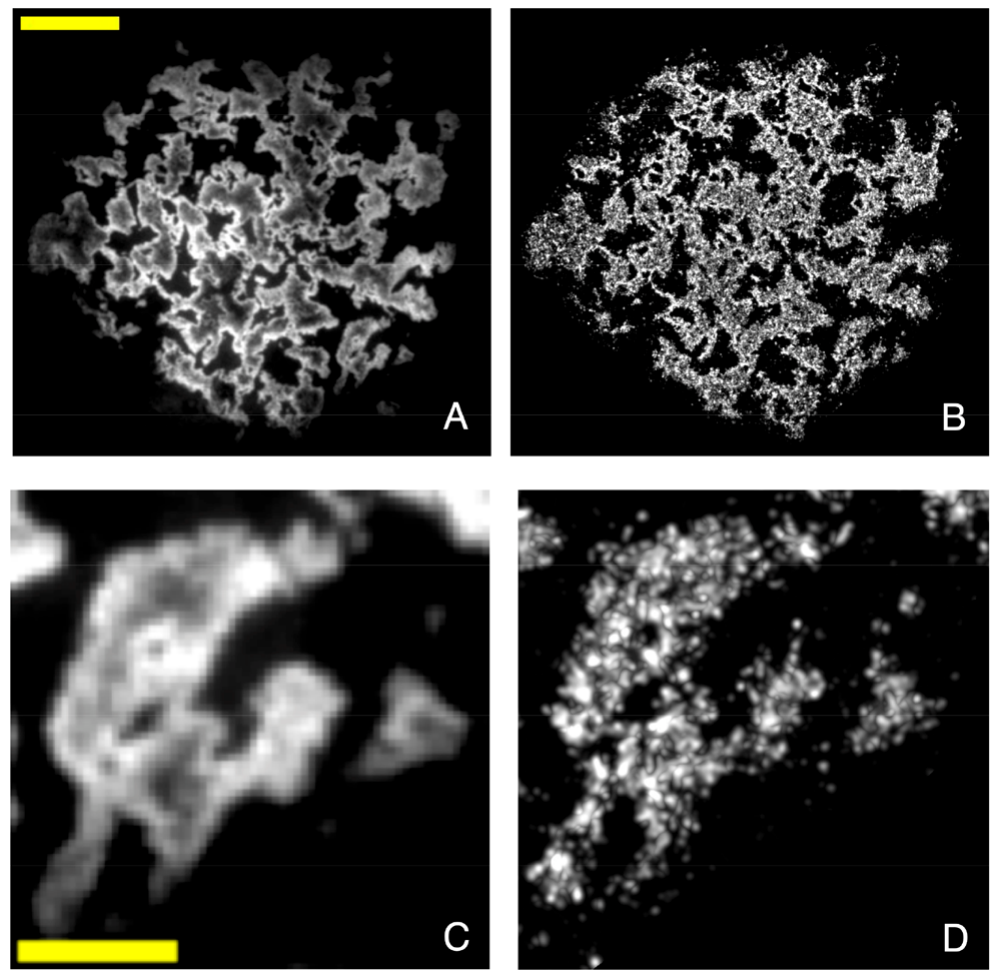
Fluorescence intensity images of the contact area between a rough PMMA sphere pressed against a DCDHF-functionalized glass coverslip wetted with DMSO with a force of 50 mN. (A) Diffraction-limited imaging of the real contact area of the PMMA sphere on the monolayer prior to photobleaching. High intensity corresponds to contact; low intensity corresponds to parts of the monolayer solvated by DMSO and not (fully) confined in contact. The scale bar is 10 μm. (B) Super-resolution image of the real contact of the PMMA sphere with a 56 nm resolution. The super-resolution image is reconstructed with the normalized Gaussian method, in which coordinates of each localization are represented by drawing a normalized symmetric two-dimensional Gaussian at each molecule location with a standard deviation equal to the FRC resolution. (C and D) Small parts from the real contact area of the PMMA sphere for diffraction-limited and super-resolution imaging, respectively. The scale bar is 3 μm. The pixel size of the super-resolved images is 13.5 nm.

It is noteworthy that the density of molecules in the contact area is much higher than in the noncontact images shown in Figure 1. This is because the probability of successful localization of a single molecule increases with the number of photons detected, and this is much higher for molecules in contact than for molecules that are not in contact due to the rigidochromic effect. The high contrast in the super-resolution contact images actually results from the thresholding that distinguishes contact from noncontact molecules on the basis of intensity, as discussed more extensively in section 1.3 of the Supporting Information (Figures S1D–F and S9).

To quantify the real contact area, both diffraction-limited and super-resolved images are thresholded using the adaptive average thresholding method.^44^ Thresholded images can be found in panels A and B of Figure S5. For PMMA, the real contact area values are 619 μm^2^ (*R* ≈ λ/2NA ≈ 200 nm) and 544 μm^2^ (*R* = 56 nm) for diffraction-limited and super-resolved imaging, respectively. Therefore, we conclude that the measured real contact areas of rough PS^23^ or PMMA spheres are not substantially smaller at a much higher imaging resolution. PS- or PMMA-on-glass contacts have been shown to involve large plastic deformation related to strain-hardening contact mechanics, which precludes structure in the area of real contact at the smallest scales.^3,10^ Comparison of panels A with B of Figure 2 suggests that within the large contact patches, small noncontact areas are present, but this has a negligible effect on the overall real contact area.

Plastic deformations dominate the contact formation for the PMMA-on-glass system. An important question is whether this would also be the case for stiffer materials such as glass. Thanks to our super-resolution imaging method which improves lateral resolution >3 times compared to diffraction limited optical imaging, we can test this experimentally. To do so, a rough glass sphere (0.5 mm in diameter) is pressed on the DCDHF functionalized glass surface with the same normal load of ∼50 mN and with the same experimental conditions as described previously for the PMMA sphere. Figure 3 shows the diffraction-limited (Figure 3A) and super-resolved (Figure 3B) images of the real contact area of the glass sphere with a 59 nm lateral resolution. The molecular density is calculated as ∼925 molecules/μm^2^, which yields a resolution of ∼67 nm according to the Nyquist–Shannon theorem, which agrees with FRC analysis (Figure S6D). In contrast to the polymer contact, here many isolated contact patches are observed that are smaller than the diffraction limit (Figure 3C vs Figure 3D). The areas of real contact are 61 and 26 μm^2^ for diffraction-limited and super-resolution imaging, respectively. The 3-fold improvement in lateral resolution, from diffraction-limited *R* ≈ 200 nm to super-resolved *R* = 59 nm, decreases the area of real contact by a factor of ∼2.4 (Figure S6). Theoretically, with an increase in the duration of the measurements, the resolution could be further improved to <10 nm, on the basis of the initial surface density of ∼80 000 molecules/μm^2^. However, waiting for the recovery of a larger fraction of molecules will take inconveniently long, and as shown below, the currently obtained resolution is adequate for the analyses of the contacts discussed in this work.

**Figure 3:**
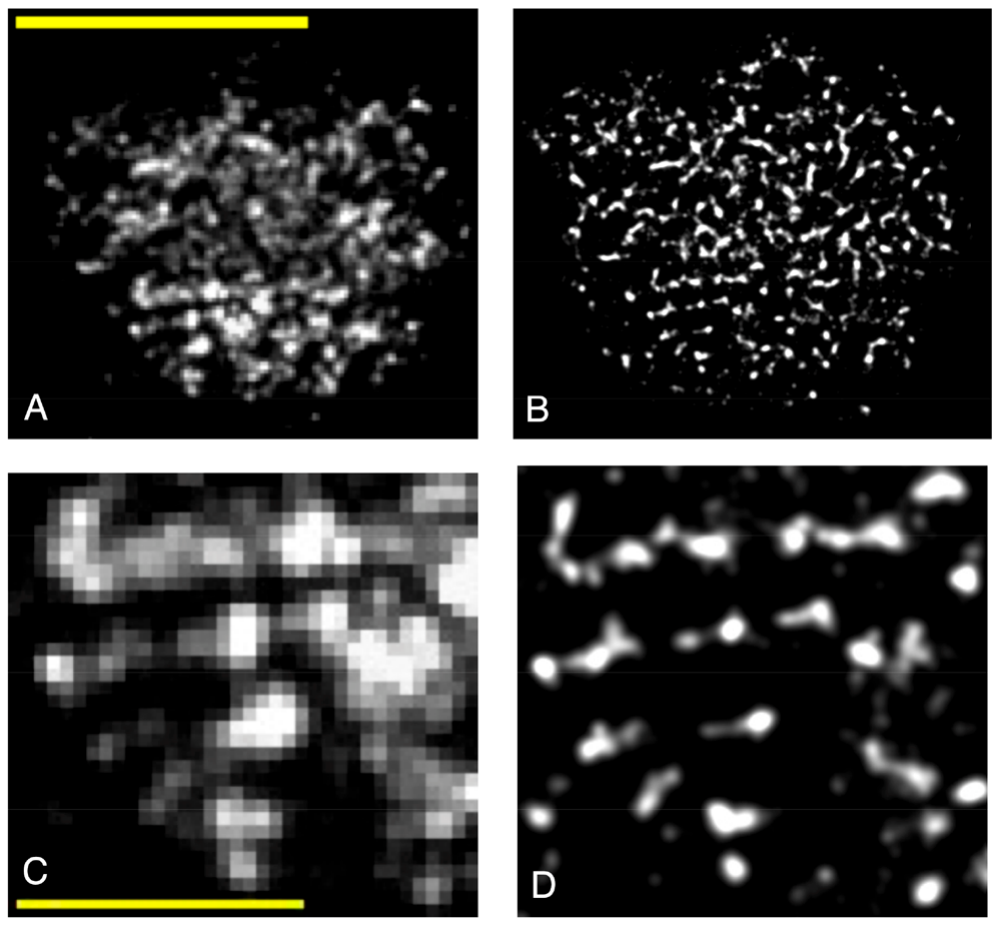
Fluorescence intensity images of the contact area between a rough glass sphere pressed against a DCDHF-functionalized glass coverslip wetted with DMSO with a force of 50 mN. (A) Diffraction-limited imaging of the real contact area of the glass sphere on the monolayer prior to photobleaching. The scale bar is 10 μm. (B) Super-resolution image of the real contact of a glass sphere at 59 nm resolution. The super-resolution image is reconstructed with the normalized Gaussian method, in which coordinates of each localization are represented by drawing a normalized symmetric two-dimensional Gaussian with a standard deviation equal to the FRC resolution. (C and D) Small parts of the real contact area of a glass sphere for diffraction-limited and super-resolution imaging, respectively. The scale bar is 2 μm. The pixel size of super-resolved images is 13.5 nm.

The image in Figure 3B shows that indeed stiffer contact interfaces contain much more small scale structure (smaller than the diffraction limit), which requires super-resolution imaging. While the super-resolved images reveal glass contact structure at scales that were previously inaccessible, the question of whether the contacts contain even more structure at unresolved length scales can still be raised. To assess this, we perform boundary element method (BEM)^17^ contact calculations based on the topography of the glass sphere surface. The glass sphere topography was measured using tapping mode atomic force microscopy. Material properties Young’s modulus, Poisson ratio, and hardness used as input for the contact calculations are listed in Table S1. In the calculations, the experimental normal load of 50 mN is applied to bring the relatively rough sphere surface (Figure 4A) in contact with a flat and smooth countersurface. The contact pressure distribution and real contact area could thus be calculated (see the Supporting Information for further details and Figure S7).

**Figure 4:**
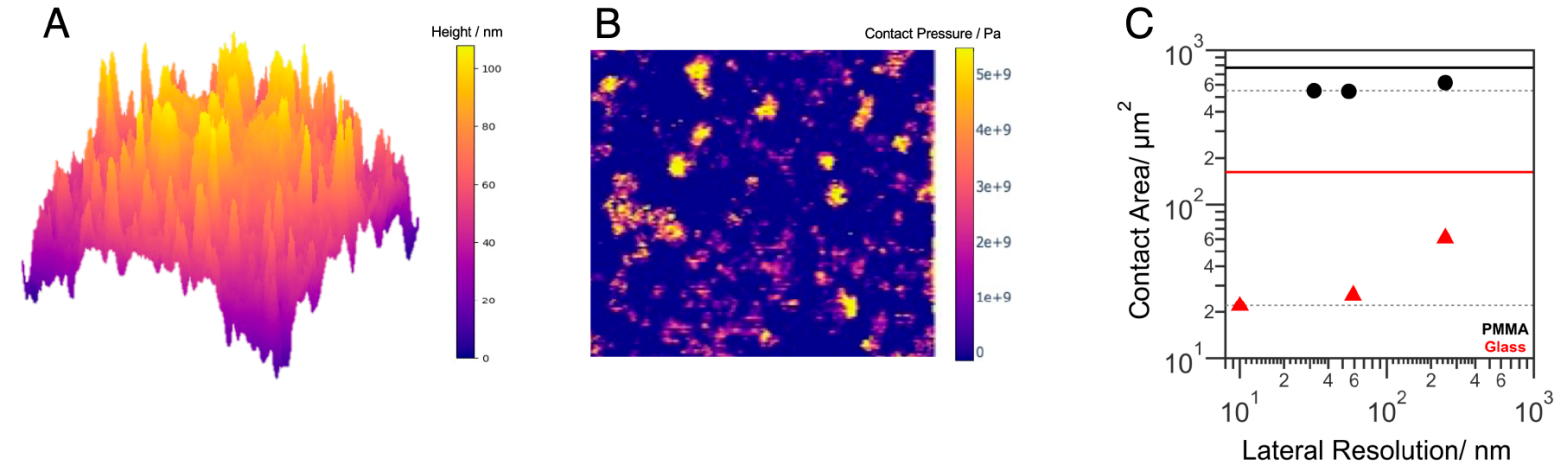
(A) Simulated glass sphere contact based on the experimental AFM topography. The area of the image is 10 μm × 10 μm. The sphere curvature is added on the basis of the dimensions of the experimental sphere (see the Supporting Information). (B) Part of the contact pressure distribution map of the sphere given in panel A as a result of contact simulation. The area of the image is 5 μm × 5 μm. (C) Contact area as a function of lateral resolution calculated for rough PMMA and glass spheres. Contact area values obtained experimentally and theoretically are plotted together in this graph, as mentioned in the text. Contact area values are plotted from lowest resolution (high resolution value) to highest resolution (low resolution value) as in the following order: experimental diffraction-limited imaging, experimental super-resolution imaging, and theoretical BEM simulation (BEM). The black and red solid lines are the contact areas from calculations with Hertz model for smooth PMMA and glass spheres, respectively.

The real contact area predicted by the BEM simulation, with a lateral resolution of ∼10 nm, for the rough glass sphere is 22.0 ± 0.2 μm^2^, which is close to the experimental super-resolved real contact area presented in Figure 3B. This result strongly suggests that the contact structure is resolved by using our super-resolution technique. The root-mean-square (RMS) surface slopes based on the AFM topographies are in the range of 0.13–0.22 for the rough glass sphere. According to Persson’s theory,^20^ an RMS slope of 0.03 is sufficient to reach contact pressures on the order of ∼6 GPa, the expected hardness of glass. Therefore, slopes found for the glass spheres used in this study are sufficiently high for plastic deformations to occur, which is also supported by the contact pressure distribution maps obtained from BEM calculations; the contact pressure on some asperities is observed to be equal to the hardness of the glass (Figure 4B). By combining all of the real contact area results obtained experimentally and theoretically in Figure 4C, we observe that the stiffer glass contacts contain structural features smaller than those of the plastic contacts. The small difference in the glass real contact area values obtained from the BEM calculations and super-resolution imaging might reflect the fact that there are small individual contact patches that are smaller than the resolution and overestimated in size. However, most of the area of real contact consists of larger patches of which the contact area can be reliably measured by super-resolution imaging. It is worth noting that the length scales that cannot be accessed by AFM can also play a role in the real contact area of glass spheres.^45^

In conclusion, we present a method for super-resolution measurement of the real contact area of rough sphere-on-flat plastic and glass contacts. The “contact sensor” is prepared by partial photobleaching of a monolayer of the rigidochromic DCDHF dye until only single molecules are visible in the fluorescence image with a nearly stable molecular density. The blinking dynamics of the single molecules allow us to apply localization microscopy for super-resolution. Because of the high number of photons per blinking event, high survival fraction, and low duty cycle, our approach provides an ideal set of properties for super-resolution microscopy via SMLM. Under contact, due to the rigidochromic effect, the number of photons is further enhanced, which improves the contrast because the contacted molecules are more efficiently localized than those in the background. We applied this method to visualize the mechanical contacts between rough PMMA and glass spheres on a DCDHF-functionalized glass coverslip. In comparison to conventional diffraction-limited imaging (*R* ≈ 200 nm), the lateral resolution was improved >3-fold with our super-resolution imaging approach. This method allows imaging of the contact patches in multi-asperity rough contacts in great detail. The achieved improvement provides an important observation for contact mechanics. For the soft PMMA sphere, the imaging resolution does not affect the real contact area much due to the presence of large plastic deformations, and therefore, the real contact area can be resolved by regular microscopy. For the much harder glass spheres, the >3-fold improvement in the resolution decreases the real contact area 2.4-fold because many of the contact patches are smaller than the diffraction limit due to the higher hardness. For both PMMA and glass spheres, the real contact area is observed to converge at a particular resolution due to the plastic deformations, but at different length scales as proposed theoretically and verified experimentally by this study. Theoretical efforts have only scarcely been connected to multi-asperity contact interface observations. Plasticity is challenging to accurately account for at the nanoscale but can at the same time be critical in defining the friction behavior of an interface.^46^ Our methodology opens new opportunities for quantitative comparison of plasticity models to experimental observations of contacts, contributing to a predictive understanding of friction.

## Data Availability

Data underlying the paper are available at https://doi.org/10.21942/uva.24560293.

## Supporting Information Available

Experimental materials and methods, including the preparation of dye-functionalized glass coverslips, the experimental setup, AFM measurements of glass spheres, SMLM analysis, characterization of SMLM properties of the dye, additional measurements (Figure S1–S9), and Python code for simulating the rough sphere from AFM topography (PDF)
