# Supplementary material for: Resolving Multi-Asperity Contacts at the Nanoscale through Super-Resolution Fluorescence Imaging
Source: J Phys Chem Lett. 2024 Feb 12;15(7):1936–42. doi: 10.1021/acs.jpclett.3c02799 (PMC10895690; doi:10.1021/acs.jpclett.3c02799)
Supplement: Supplementary file 1 — jz3c02799_si_001.pdf [file jz3c02799_si_001.pdf]

Supporting Information

Resolving Multi-Asperity Contacts at the Nanoscale through Super-Resolution Fluorescence Imaging

Begüm Demirkurt[1], Dina Petrova[1], Dharmendar Kumar Sharma[2,†], Martin Vacha[2], Bart Weber[3,4], Daniel Bonn[4], and Albert M. Brouwer[1]*

[1] van 't Hoff Institute for Molecular Sciences, University of Amsterdam, P.O. Box 94157, 1090 GD Amsterdam, The Netherlands.

[2] Tokyo Institute of Technology, Ookayama 2-12-1-S8, Meguro-ku, Tokyo, 152-8552 Japan.

[3] Advanced Research Center for Nanolithography (ARCNL), Science Park 106, 1098 XG Amsterdam, The Netherlands.

[4] Institute of Physics, University of Amsterdam, P.O. Box 94485, 1090 GL Amsterdam, The Netherlands.

Table of Contents

1. Methods S3

1.1 Preparation of Glass Coverslips with Immobilized DCDHF dye S3

1.2 Experimental Setup S3

1.3 Single Molecule Localization Analysis S3

1.4 Single Molecule Localization Properties of Single DCDHF Molecules in Partially Bleached Monolayer S5

1.5 Atomic Force Microscopy Measurements of Glass Spheres and Analysis ... S8

1.6 Contact Mechanics Calculations and Boundary Element Method (BEM) Contact Simulations S9

1.7 Materials S11

2. Additional Measurements S12

3. Python Code S20

4. References S22

Raw data that support the findings of this study are available on figshare: https://doi.org/10.21942/uva.24560293

1. Methods

1.1 Preparation of Glass Coverslips with Immobilized DCDHF dye

DCDHF immobilized glass coverslips were prepared according to the two-step procedure reported in Reference 1.

1.2 Experimental Setup

The contact was established by pressing a PMMA/PS/glass sphere by attaching it to the Anton–Paar DSR 301 rheometer as specified throughout the text and in References 1 and 2. A home-built open frame widefield/TIR epifluorescence microscopy set-up was used to image the contact interface. A sapphire LP CW (Coherent) laser was used at 488 nm wavelength to illuminate the surfaces. In all experiments, the power density was kept the same at 1 $kWcm^{-2}$. A 535/50 emission filter and a 488 nm notch filter were used in the setup. The objective used was an Olympus UAPON 100X, 1.49 NA. The light was recorded on the sCMOS camera Hamamatsu C11440 either by 50 ms or 100 ms per frame as specified in the main text. For the results that are presented in Figure 1, no pixel binning was applied, however, for the mechanical contact experiments, 2 × 2 pixel binning was used.

Spectra were measured using a spectrograph (CLP-50LD, Bunkou Keiki) coupled to an Ixon Andor EM CCD 8987 camera. An integration time of 10 ms was used. EM gain was 0 and 60 for bulk spectra and single-molecule spectra, respectively. The spectra were smoothed with a binomial smoothing filter (smoothing parameter set to 7) in Igor Pro software.

1.3 Single Molecule Localization Analysis

Single molecule localization for super-resolution analysis was done by using the ThunderSTORM plug-in on ImageJ.[3] The following camera setups were used in the analysis: 1 pixel = 68 nm, photoelectrons per A/D counts = 0.49, Base level (A/D counts) = 100 for 1×1

binning, 400 for 2×2 binning). Wavelet filter (B-spline) was selected in image filtering (3rd order, scale 2.0). Local maximum method with 8–neighborhood connectivity was chosen for approximate localization of molecules. In the local maximum method, a single molecule intensity threshold is determined by the standard deviations of the intensities in the filtered images. Integrated PSF model for symmetric 2-D Gaussian function was selected for subpixel localization of molecules with fitting radius of 3 pixels by using the Maximum-likelihood model with initial 1.6 pixels sigma size. Next, molecules with (nearly) the same coordinates were merged in ThunderSTORM with the following parameters: maximum distance = average localization uncertainty, maximum frames per molecule = 0, maximum off frames = total number of frames – 1. Then, molecules were filtered based on their sigma widths with the following requirement: 1 nm < sigma < 300 nm. After this step, the super-resolution image was reconstructed with coordinates of molecules via Normalized Gaussian representation in which the sigma width was equal to the calculated Fourier Ring Correlation (FRC) resolution mentioned in the next paragraph.[4] For PMMA sphere-on-glass contact, 20000 frames were recorded from the end of 15 min to 2 hours of bleaching and analyzed fully. For glass-on-glass contact analysis, 3556 frames were recorded at the end of an hour of photobleaching.

The resolutions of super-resolved images were obtained by using Fourier Ring Correlation analysis as given in Reference 4. First, localizations were divided into two statistically independent data sets (even frames and odd frames) on ThunderSTORM[3] by the following operation: for splitting out only even frames, setting filter on ThunderSTORM[3] to *frame%2=0*. Then, using Normalized Gaussian visualization along with average lateral uncertainty value, the super-resolution images are reconstructed. Afterwards, the same procedure is repeated for the odd frames by setting the filter to *frame%2=1*. For resolution determination of super-resolution images of

mechanical contacts, the FRC plug-in in ImageJ (BIOP)[5] was used, which uses the aforementioned two sub-super-resolution images as input and seeks for the lowest distance at which the correlation of these two subsets is met.[4] A fixed value of 0.143 is used as threshold for the correlation. The block-wise FRC calculation was applied by using NanoJ-SQUIRREL plug-in on ImageJ with default settings (Figure S5C and Figure S6C).[5]

Real contact area values were obtained by applying the following image processing steps using ImageJ: for the diffraction-limited imaging, an Adaptive Mean Thresholding method[6] was applied after background subtraction. The search radius was selected as the radius of the smallest resolvable contact patch. For super-resolution images, the same thresholding method was applied. By overlaying the real contact areas and the single molecule images, the number density of molecules in the contact regions are calculated as ~1100 molecules per μm^2 for the PMMA sphere-on-glass contact and ~925 molecules per μm^2 for the glass sphere-on-glass contact.

1.4 Single Molecule Localization Properties of Single DCDHF Molecules in Partially Bleached Monolayer

Here, we report the single-molecule localization properties of the surface-bound single rotor molecules to characterize them as a single-molecule localization microscopy (SMLM) probe. The chemistry behind the single-molecule fluorescence blinking of DCDHF rotor molecules at constrained, glass interfaces will be discussed in more detail in a future paper. For the characterization of the probe, we analyze the following parameters which predominantly determine the final resolution in the super-resolved image: molecular density, average number of photons per blinking cycle, survival fraction, and duty cycle.[7] The molecular densities of the blinking monolayer at glass-air interface (without mechanical contact) and at the end of 2h of bleaching are found as ~100 molecules per μm^2 from which the theoretical resolution of ~200 nm

is calculated by using the Nyquist-Shannon sampling theorem. However, when surface-bound molecules were constrained more by a thin layer of spin-coated PMMA film on top of them, a 5 times denser blinking monolayer was obtained along with ~90 nm Nyquist resolution. The experimental resolution of super-resolved mechanical contact images, however, is found much higher than these values. The main reason for this difference lies in the different density of fluorescent molecules that the monolayer possesses with and without mechanical contact. The molecular densities in the contact regions are ~1100 molecules per μm^2 for the PMMA sphere-on-glass contact and ~925 molecules per μm^2 for the glass sphere-on-glass contact, significantly denser than in the absence of mechanical contact. This denser blinking monolayer allows the theoretical resolution of ~61 nm for the PMMA contact and ~67 nm for the glass contact according to the Nyquist-Shannon theorem, which are close to the results of the FRC analysis as reported in the main text. The average number of photons per blinking cycle (ON period duration) (Figure 1F in the main text) determines how precise the locations of single molecules can be found. The calculated average number of photons per blink is well in the range of ~8000 – 12000 photons per event required for a good localization probe along with a reasonable average localization precision of 13 nm for the molecules at glass-air interface.[7] However, when the surface-bound molecules are in contact with a rough sphere, the average localization precision is found to be in the range of 20 – 25 nm. This change might be due to the presence of mechanical drift in the experimental setup. Even though the super-resolution images are corrected by the cross-correlation method implemented in ThunderSTORM,[3] the effect of mechanical drift that is faster than our imaging speed would still be present. The survival fraction, which shows the active emitter concentration throughout the experiment, is an important parameter to reveal the control over molecular density. Molecular density is the main limitation of all SMLM approaches, since the locations of molecules

is the only information used for surpassing the diffraction limit. As shown in Figure S1B, the survival fraction after 2 hours under contact was found as > 90%, indicating a stable density of single molecules and an ideal behavior of a good localization probe.[7] Lastly, the duty cycle, which is the ratio between the number of molecules in ON state and OFF state, is important for the localization success. Probes with a too high duty cycle have too many molecules in the ON state, leading to overlapping emission profiles of single molecules, which precludes high localization precision. For our probe, a duty cycle of 0.001 is estimated as presented in Figure S1C, which again brings about good localization probe characteristics (duty cycle < 0.003).[7] In the light of these results, we conclude that DCDHF molecules in the partially bleached monolayer satisfy the criteria for super-resolution imaging fluorophores via SMLM remarkably well. Moreover, these satisfactory results for the probe are achieved with a relatively simple experimental procedure as described in this paper, which is a desirable combination for any SMLM approach.

As mentioned in the main text, we spectroscopically show that the surface-bound single rotor molecules retain their contact sensitivity after prolonged exposure and in the presence of DMSO (Figure S2A and S2B). To verify this contact sensitivity also *in situ* for our method, we perform another contact experiment with a PS sphere on glass, where after first contact formation, two cycles of "bleaching – lifting the contact – restoring the same contact" experiments are performed by using the same DCDHF functionalized glass surface and the same PS sphere. As shown in Figure S4A and S4C, the number of fluorescent molecules slowly increases after switching off the laser for 1 hour. Remarkably, the recovery is strongly enhanced by raising the sphere from the contact. After 1 minute the sphere is pressed against the coverslip again so that the contact image can be recorded again, and we observe almost full recovery of the fluorescent monolayer (Figure S4D and S4F). Therefore, we can conclude that the surface-bound rotor molecules retained their

contact sensitivity, and molecular fluorescence properties of the markers that recover after being photobleached have not been altered by prolonged exposure to the intense laser light.

As mentioned above, the number density of single molecules in contact is much higher than in the reference image of the cover slip (Fig. 1). The reason is that the processing of the data favors the detection/localization of single molecules with a higher intensity, that is: molecules in contact have a higher probability of being identified because of their higher intensity. This gives rise to the contrast in super-resolution imaging of the contacts. The non-contacted molecules which are solvated by DMSO fluoresce weakly, many with intensities lower than the intensity threshold used in the first stage of single molecule localization as described in Section 1.3. For example, for a non-contact sample where the monolayer is covered with DMSO this threshold is ~3 times smaller than for the PMMA contact image. This means that in the contact image many of the non-contact molecules are not identified because they fall below the threshold. Some non-contacted molecules are still detected, but with lower intensities than contacted molecules (Figure S1D – S1F). Moreover, many of these have large sigma widths and are removed in the post-processing stage as we described on page S4 in section 1.3. We illustrate the effect of this last filtering step in Figure S9, which shows that many non-contacted molecules (located mostly in the out-of-contact zones) are eliminated from the raw single molecule list based on their Gaussian widths. The final density of detected molecules in the non-contact regions are found as ~10 molecules per μm^2 for glass contacts and ~40 molecules per μm^2 for PMMA contact.

1.5 Atomic Force Microscopy Measurements of Glass Spheres and Analysis

5 µm x 5 µm topographies were scanned in tapping mode using a Dimension Icon atomic force microscope (Bruker) equipped with RTESPA-150 probes (Bruker). Images of 512 x 512 pixels were recorded at a scan velocity of 0.2 lines per second. AFM topographies are analyzed with Gwyddion Software.[8] For all AFM topographies, the following analysis workflow is applied: Lines

on AFM topography images due to scanning issues are removed by applying the 'align rows' function in the software by taking the line directions into account (horizontal or vertical) and with trimmed mean of differences (4.5%). Then, the spherical background is removed by using second degree polynomial in both directions. Finally, the data is levelled by mean plane subtraction and minimum is shifted to zero for better representation of the height values.

1.6 Contact Mechanics Calculations and Boundary Element Method (BEM) Contact Simulations

In Figure 4C in the main text, theoretical contact area values for PMMA and glass spheres are calculated by using the Hertz theory as mentioned in the main text. Briefly, the theory assumes that the two contacting surfaces are smooth (neglects the roughness) and contact is elastic.[9]

The Hertz equation. $\boldsymbol{A_{contact}}$ is the apparent area of the contact between a sphere (diameter D) and a flat surface pressed against each other with normal load of $\boldsymbol{F_{Normal}}$. $\boldsymbol{E^*}$ is the effective Young's modulus and $\boldsymbol{\nu}$ are Poisson ratios of the materials in contact.

$$A_{contact} = \pi \left(\frac{3R}{4E^*}\right)^{\frac{2}{3}} (F_{normal})^{\frac{2}{3}} \qquad \frac{1}{E^*} = \frac{1-\nu_1^2}{E_1} + \frac{1-\nu_2^2}{E_2}$$

Table S1. Parameters for the Hertz model calculations.

E_{PMMA} (GPa)	E_{Glass} (GPa)	ν_{PMMA}	ν_{Glass}	F_{normal} (mN)
2.5	64	0.3	0.2	50

In the main text, the theoretically expected root-mean-square (RMS) surface slope corresponding to a contact pressure equal to the hardness of glass is calculated by using Persson's theory.[10] According to Persson theory, the contact pressure is proportional to the RMS surface slope. This theory takes roughness into account as adding power spectral density (PSD) of the deformed surface into the theory and gives exact solutions for full contact conditions via the half-space approximation.

Persson's contact mechanics theory. $\boldsymbol{m_2}$ is the mean square surface slope and $\boldsymbol{\Lambda}$ is the numerical factor for Persson's theory.

$$A_{contact} = \Lambda \left(E'\sqrt{2m_2}\right)^{-1} F_{normal}$$

$$E' = \frac{E}{1-\upsilon^2} \qquad \Lambda = \sqrt{8/\pi}$$

As the contact pressure must be limited by the hardness (H) of the materials, we can set an upper limit to the root mean square surface slope:

$$\frac{\mathrm{F}_{normal}}{A_{contact}} < H < \frac{E'\sqrt{2m_2}}{\sqrt{8/\pi}}$$

Young's modulus and Poisson's ratio values given for glass in Table S1 are used in this calculation. H is taken as ~5.8 GPa according to the literature.[11]

To establish a comparison between the experimental observations and theoretical predictions of the glass sphere-on-glass contact, we also conducted contact calculations employing the boundary element method (BEM), as described in the main text.[12] The simulation incorporates surface

topography data obtained through atomic force microscopy (AFM) and takes the material properties and contact conditions into account. This computational approach solves the elastic equations and facilitates idealized plastic deformation to alleviate local contact pressure, reducing it to levels below the material hardness. To simulate the glass sphere, 5 μm × 5μm (512 × 512 pixels) AFM topography of the sphere is symmetrically mirrored, resulting in a topography of 10 μm × 10 μm dimensions (1024 × 1024 pixels). This dimension is needed to maintain the similar image sizes with the experiments. Afterwards, the spherical curvature is added to the new, mirrored topography with the Python code given in Section 3, by using the same geometrical dimensions of the experimental sphere. This procedure is demonstrated in Figure 4A and Figure 4B in the main text. The same procedure is applied to 10 different AFM topographies recorded from the surface of the glass spheres. All BEM simulations are run through the cloud based TriboSolver software given in the Reference 13. For BEM simulations of rough glass spheres-on-glass contacts, glass parameters given in the Table S1 are used. To mimic the experiments accurately, the Young's modulus calculated experimentally by using our contact visualization method (Figure S8) is used in the simulations (E = 61.4 GPa).[2] An example simulated sphere and contact pressure distribution map for this sphere are presented in Figure S7.

1.7 Materials

Smooth PMMA and PS spheres were purchased from Cospheric (radius 500 μm). Glass spheres were purchased from Sigmund – Lindner (radius 500 μm). Glass coverslips were purchased from MARIENFELD high precision thickness 1.5H. PMMA spheres were shaken for 24 hours, using a vortex shaker, in a container with 240 grit sandpaper walls to obtain rough surfaces whereas glass spheres were roughened under same conditions for 1 hour.

2. Additional Measurements

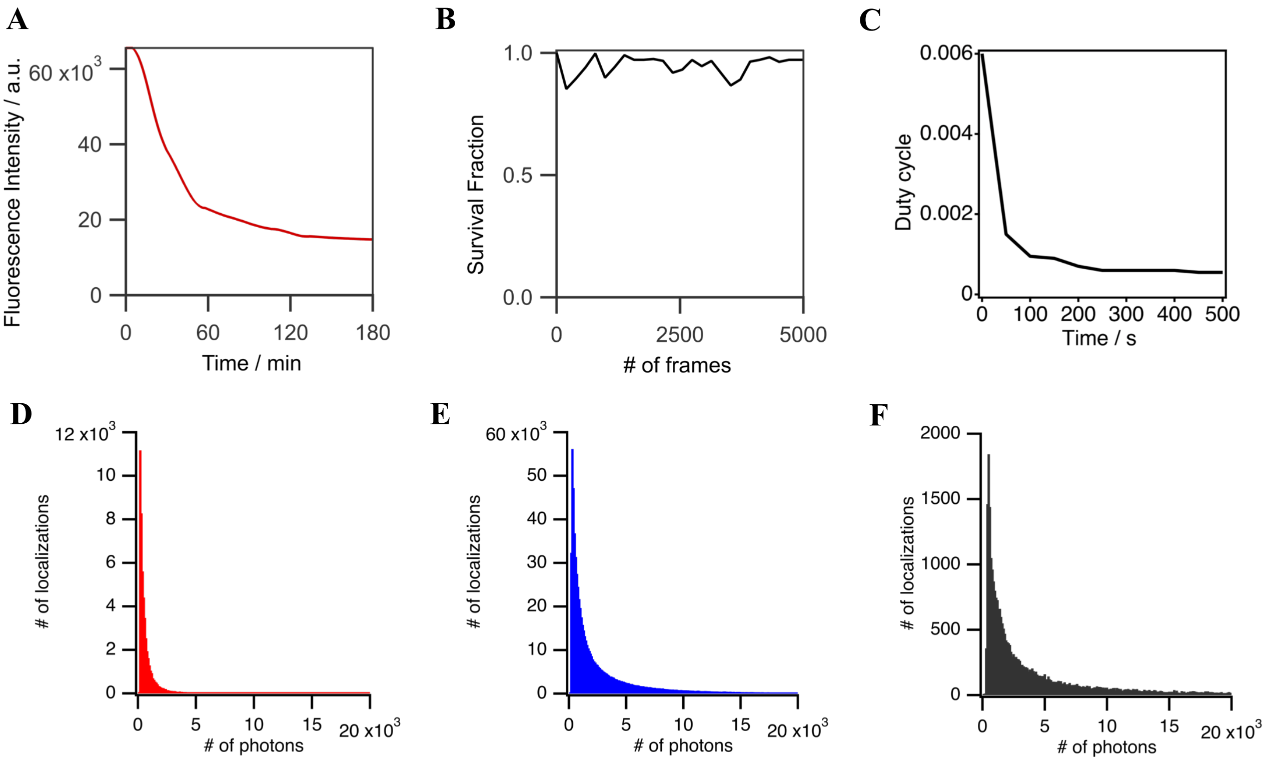

Figure S1. A. Example of bleaching during the experiment. Fluorescence intensity (number of counts) from an area of 36 μm^2 during photobleaching of DCDHF functionalized glass with continuous laser irradiation (1 kW cm^{-2}). **B.** Survival fraction and **C.** duty cycle of blinking monolayer under mechanical contact as a function of recording time. **D., E.** and **F.** Histograms of average number of photons per molecule per blinking cycle (ON-time period) for DMSO-solvated surface bound-DCDHF molecules (non-contacted) and molecules that are under contact with PMMA and glass spheres, respectively.

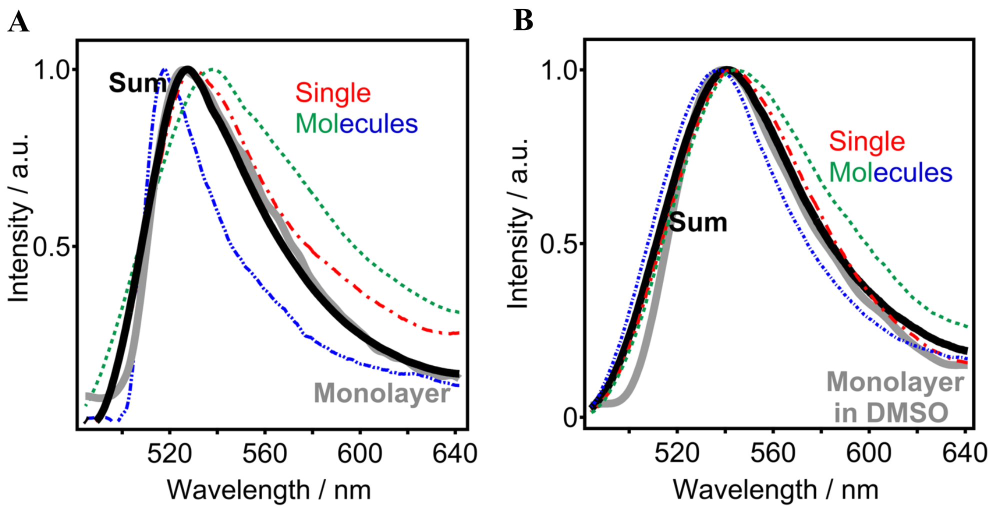

Figure S2. Red, green and blue: fluorescence spectra of single molecules remaining on the coverslip after bleaching. Black: sum of spectra of 10,000 molecules measured at the coverslip. Grey: initial spectrum of the monolayer before bleaching. **A.** Monolayer of DCDHF on the coverslip without added solvent. **B.** Monolayer of DCDHF on the coverslip solvated by DMSO. Excitation wavelength 488 nm.

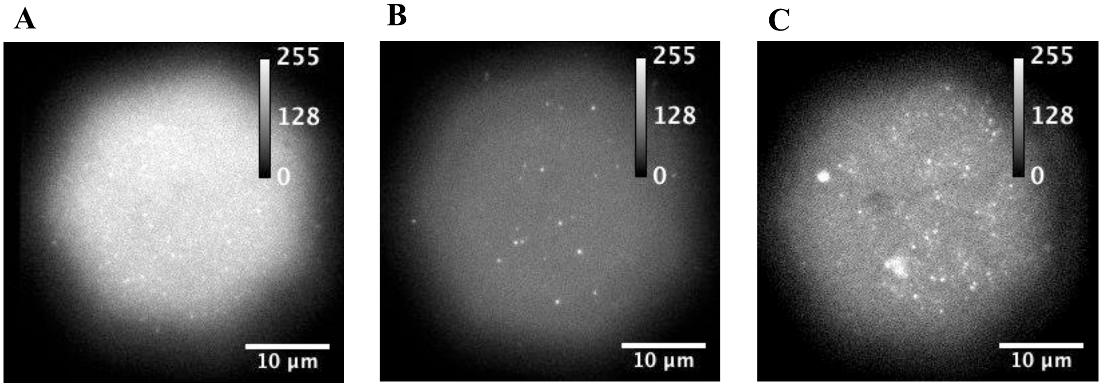

Figure S3. Fluorescence intensity images of: **A.** "Blinking" dry monolayer. **B.** "Blinking" monolayer immersed in DMSO. **C.** "Blinking" monolayer in contact with PS sphere.

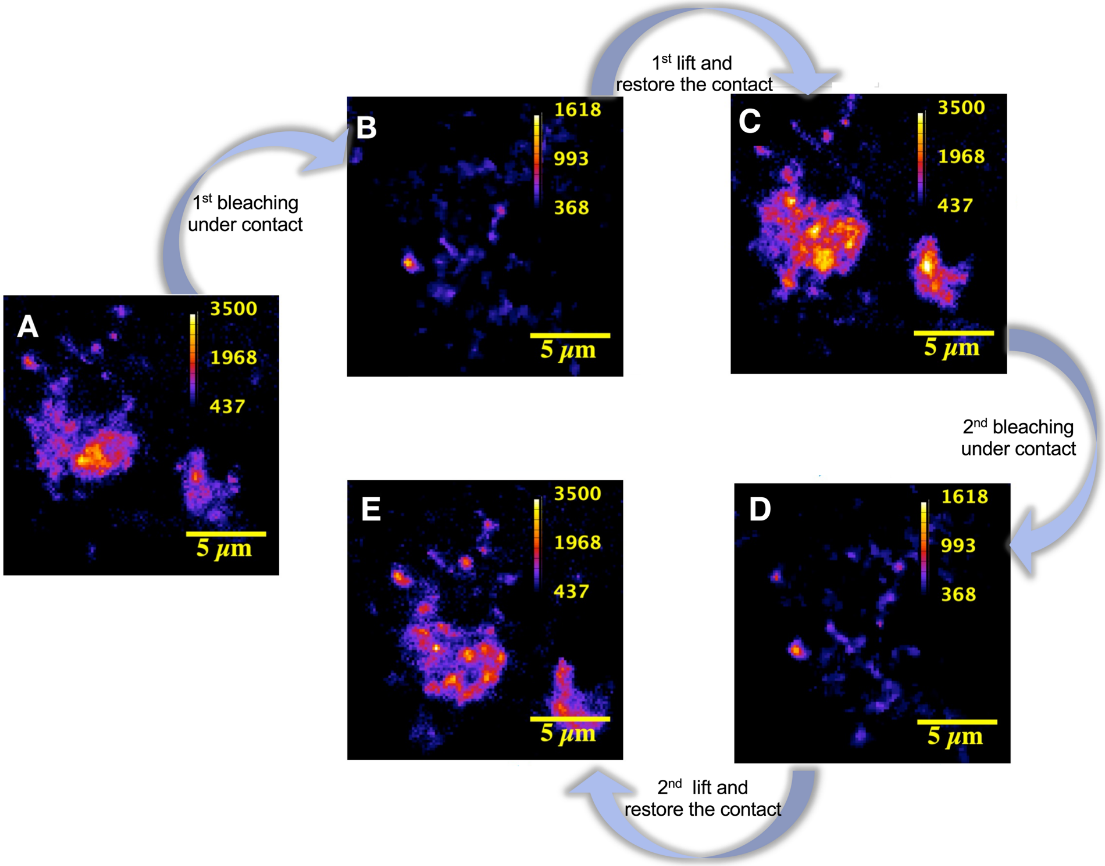

Figure S4. Fluorescence images of contact area between a rough PS sphere and a DCDHF monolayer. **A.** Original contact, diffraction limited image. **B.** Bleached contact, single-molecule level image. **C.** Contact after waiting for 1 h off-laser under contact, then removing the sphere for 1 minute, and pressing it onto the monolayer again. **D.** Bleached contact **C**, single-molecule level image. **E.** Contact after waiting for 1 h off-laser under contact, then removing the sphere for 1 minute, and pressing it onto the monolayer again.

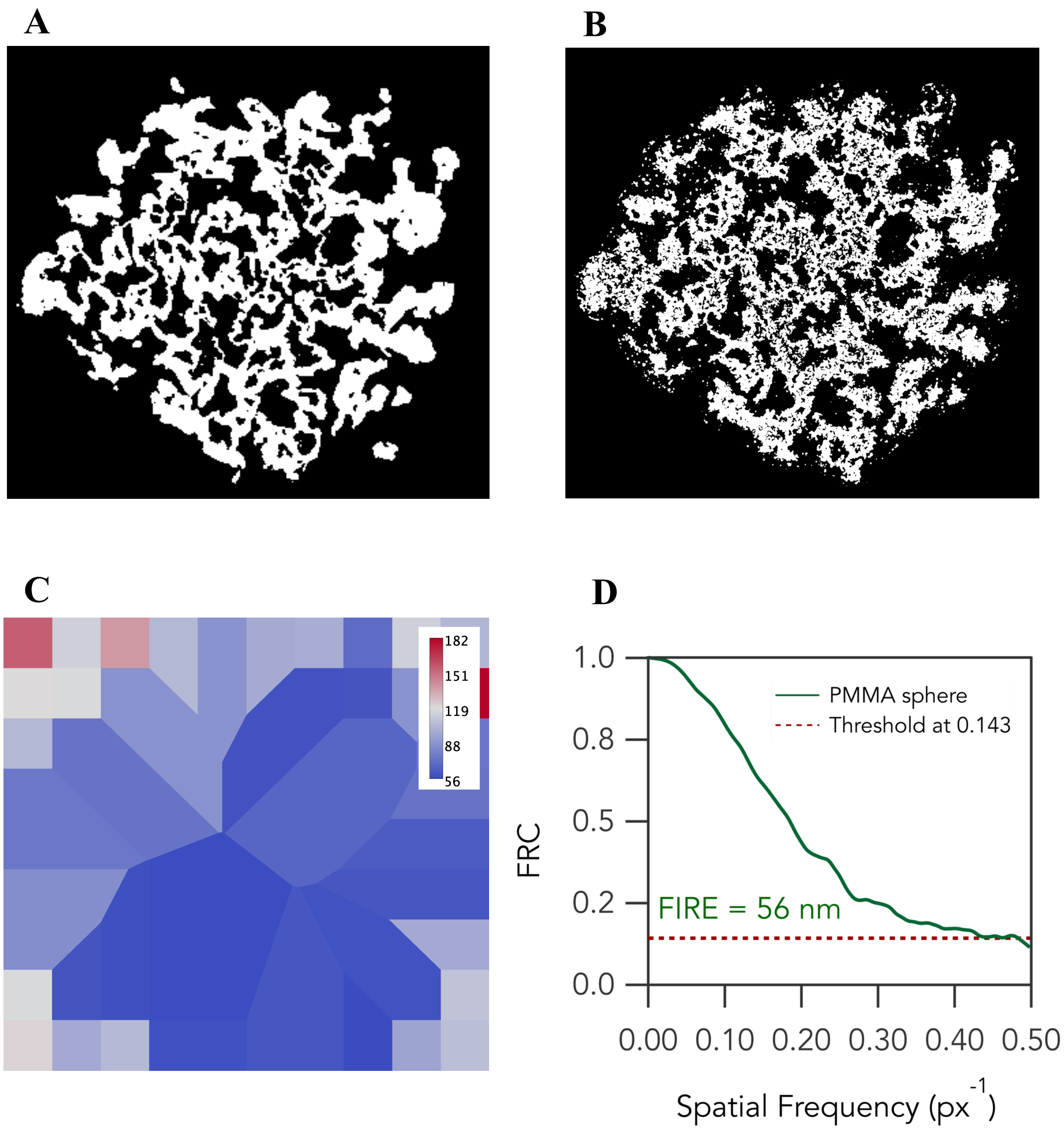

Figure S5. **A.** Thresholded Figure 2A in the main text. **B.** Thresholded Figure 2B in the main text. **C.** FRC map of the super – resolution image in Figure S5B. Calibration bar is in nm as unit, which shows FRC value distribution over the image. **D.** FRC curve of Figure 2B in the main text, which gives the average FRC resolution of ~56 nm.

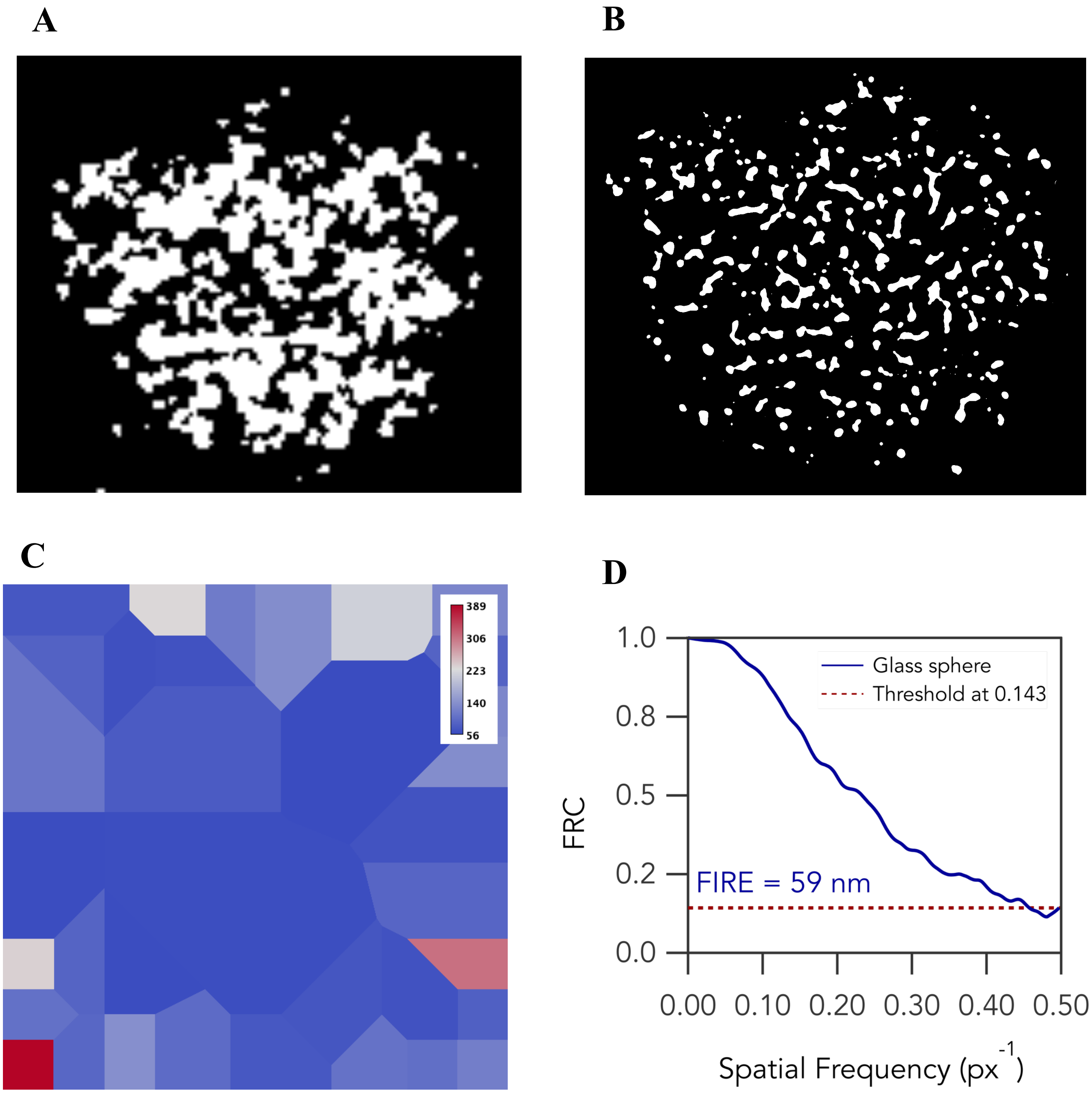

Figure S6. A. Thresholded Figure 3A in the main text. **B.** Thresholded Figure 3B in the main text. **C.** FRC map of the super – resolution image in Figure S6B. Calibration bar is in nm as unit, which shows FRC value distribution over the image. **D.** FRC curve of Figure 3B in the main text, which gives the average resolution of ~59 nm.

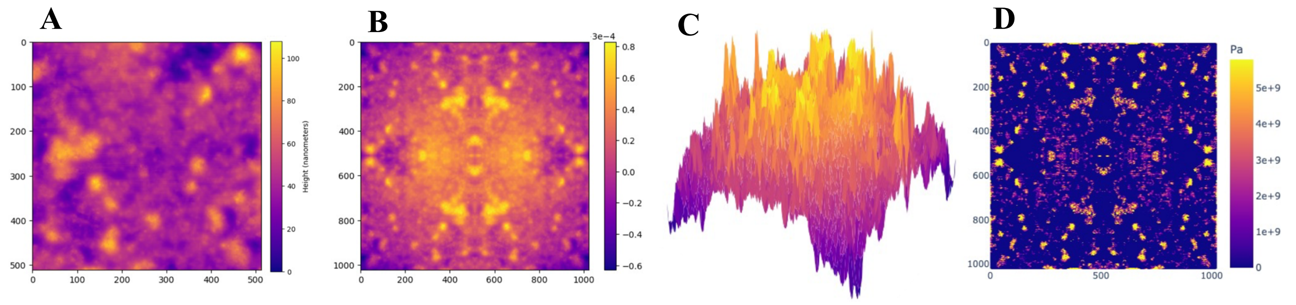

Figure S7. A. The AFM topography of the glass sphere. The image is 5 µm x 5 µm. The sphere curvature was subtracted from the AFM topography to highlight the surface roughness. **B.** Simulated glass sphere in 2-D in which AFM topography data in **(A)** is used. **C.** 3-D representation of the simulated sphere in (**B**). **D.** Contact pressure distribution map of the sphere given in **(B)** (and **(C)**) as a result of contact simulation with 23 µm^2 real contact area.

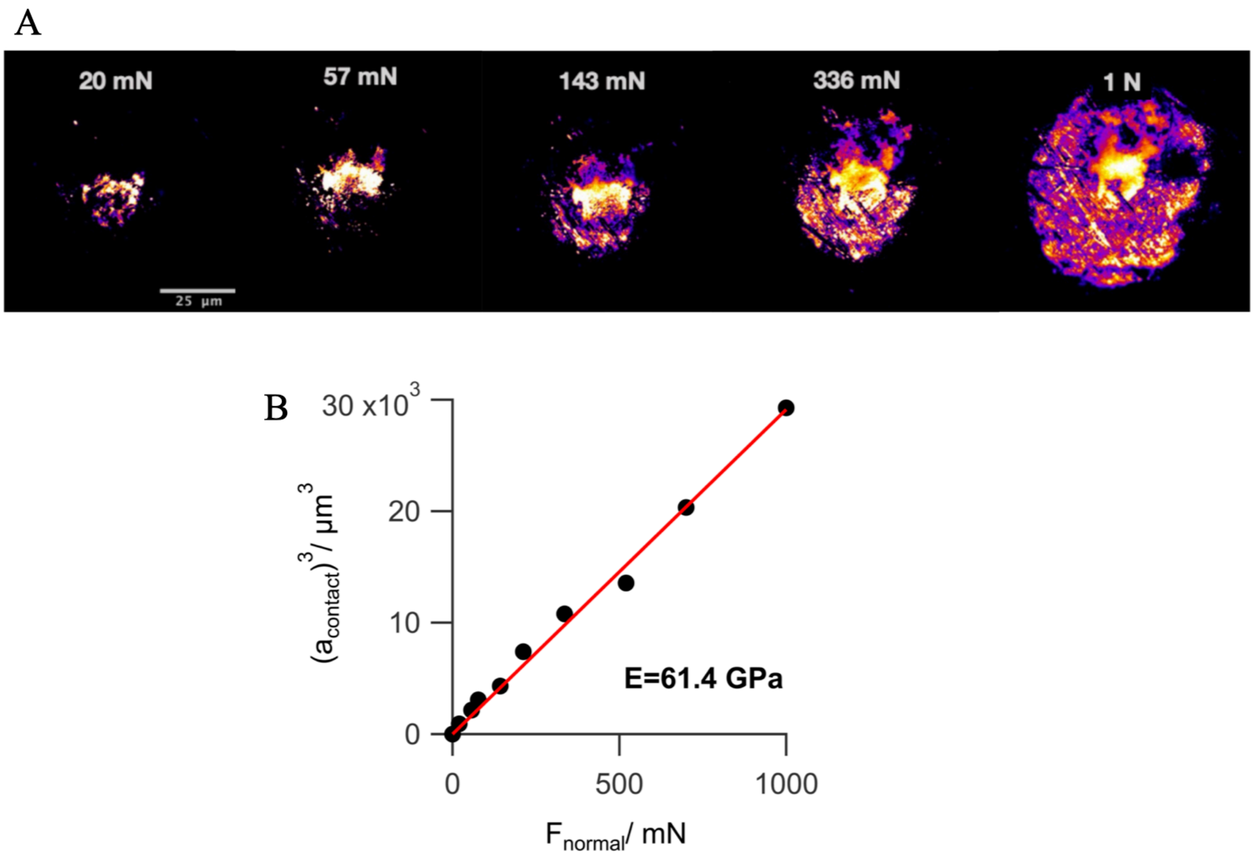

Figure S8. A. Diffraction-limited imaging of contact area of a rough glass sphere made of same type of glass used in this study under increasing normal load. **B.** Radius of contact area observed in the fluorescence images shown in **(A)** as function of the normal force according to the Hertz equation.

A B

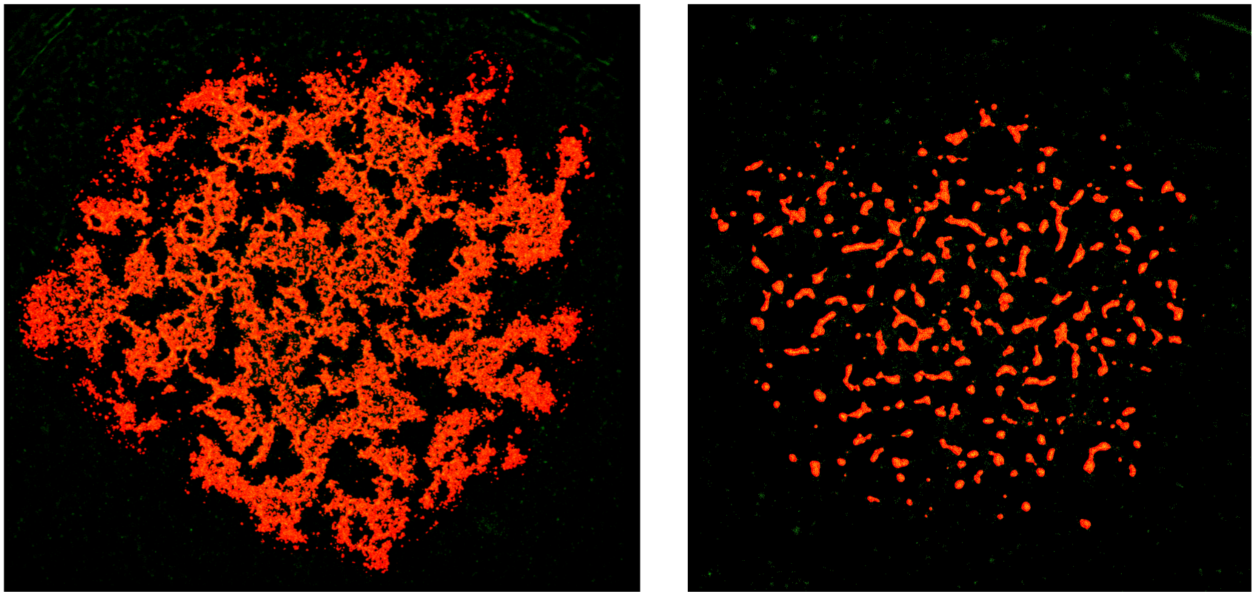

Figure S9. Merged images of contacts (**A:** PMMA; **B:** glass) showing positions of all single molecules after the localization, and the contact area (red). Molecules that are within the contact zone are shown in yellow and the molecules that were filtered out in the post-processing based on their width are shown in green. For further information, see section 1.3 on page S4.

3. Python Code

```
import numpy as np
import matplotlib.pyplot as plt

# Load the initial AFM height data from a .txt file
afm_data = np.loadtxt('yourAFMdata.txt')

# Mirror the AFM height data both horizontally and vertically:stitching
mirrored_data = np.flip(afm_data, axis=0)  # Mirror vertically
mirrored_data = np.flip(mirrored_data, axis=1)  # Mirror horizontally

# Create the final bigger matrix (symmetric mirroring)
final_matrix = np.zeros((1024, 1024))

# Calculate the starting positions for each quadrant
start_x = 0
start_y = 0

# Fill in the quadrants of the final matrix
final_matrix[start_y:start_y + afm_data.shape[0], start_x:start_x +
afm_data.shape[1]] = afm_data
final_matrix[start_y:start_y + afm_data.shape[0], start_x +
afm_data.shape[1]:] = np.fliplr(afm_data)
final_matrix[start_y + afm_data.shape[0]:, start_x:start_x +
afm_data.shape[1]] = np.flipud(afm_data)
final_matrix[start_y + afm_data.shape[0]:, start_x + afm_data.shape[1]:] =
np.flipud(np.fliplr(afm_data))

# Save the mirrored matrix to a .txt file
np.savetxt('result1.txt', final_matrix, fmt='%.4e', delimiter='\t')

# Visualize the final mirrored matrix
plt.imshow(final_matrix, cmap='jet')
plt.colorbar()
plt.show()

# Load the mirrored AFM height data
afm_data = np.loadtxt('result1.txt')

# Input: pixel size (in meters) and the sphere radius (in meters)
pixel_size =             # pixel size in meters
sphere_radius_meters =   # sphere radius in meters
```

```
# Create an empty canvas for the final 3D data (1024x1024)
final_3d_data = np.zeros((1024, 1024), dtype=np.float64)

# Calculate the center of the sphere
sphere_center_x = 512
sphere_center_y = 512

# Generate the coordinates for the sphere
y, x = np.meshgrid(range(final_3d_data.shape[0]),
range(final_3d_data.shape[1]))

# Calculate the heights of the sphere at each point in meters
sphere_heights_meters = np.sqrt((sphere_radius_meters**2) - (((x -
sphere_center_x) * (pixel_size))**2) - (((y - sphere_center_y) *
(pixel_size))**2))

# Create the final 3D data by adding the sphere curvature to the AFM data
final_3d_data = afm_data + sphere_heights_meters

# Save the final 3D data to a .txt file
np.savetxt('result3.txt', final_3d_data, fmt='%.11e', delimiter='\t')

# Visualize the final 3D data
plt.imshow(final_3d_data, cmap='jet')
plt.colorbar()
plt.show()

plt.imshow(sphere_heights_meters, cmap='jet')
plt.colorbar()
plt.show()
```

4. References

(1) Petrova, D.; Sharma, D. K.; Vacha, M.; Bonn, D.; Brouwer A.M.; Weber, B. Ageing of Polymer Frictional Interfaces: The Role of Quantity and Quality of Contact. *ACS Appl. Mater. Interfaces* **2020**, *12*, 9890-9895.

(2) Suhina, T.; Weber, B.; Carpentier, C. E.; Lorincz, K.; Schall, P.; Bonn, D.; Brouwer, A. M. Fluorescence Microscopy Visualization of Contacts Between Objects. *Angew. Chem. Int. Ed.* **2015**, *54*, 3688-3691.

(3) Ovesný, M.; Křížek, P.; Borkovec, J.; Švindrych, Z.; Hagen, G. M. ThunderSTORM: A Comprehensive ImageJ Plug-in for PALM and STORM Data Analysis and Super-Resolution Imaging. *Bioinformatics* **2014**, *30*, 2389-2390.

(4) Nieuwenhuizen, R. P.; Lidke, K. A.; Bates, M.; Puig, D. L.; Grünwald, D.; Stallinga, S.; Rieger, B. Measuring Image Resolution in Optical Nanoscopy. *Nat. Methods* **2013**, *10*, 557-562.

(5) Culley, S.; Albrecht, D.; Jacobs, C. *et al*. Quantitative Mapping and Minimization of Super-Resolution Optical Imaging Artifacts. *Nat. Methods* **2018**, *15*, 263-266.

(6) Adaptive Threshold --- ImageJ plugin - ImageJ plugins by Qingzong TSENG. Available at: https://sites.google.com/site/qingzongtseng/adaptivethreshold. (Accessed: November 15, 2023).

(7) Dempsey, G. T.; Vaughan, J. C.; Chen, K. H.; Bates, M.; Zhuang, X. Evaluation of Fluorophores for Optimal Performance in Localization-Based Super-Resolution Imaging. *Nat. Methods* **2011**, *8*, 1027.

(8) Nečas, D.; Klapetek, P. Gwyddion: An Open-Source Software for SPM Data Analysis, *Cent. Eur. J. Phys.* **2012**, *10*, 181-188.

(9) Hertz, H. On the Contact of Elastic Solids. *J. Reine Angew. Math.* **1882**, *92*, 156-171.

(10) Persson, B. N. J. Theory of Rubber Friction and Contact Mechanics. *J. Chem. Phys.* **2001**, *115* (8), 3840-3861.

(11) Boccaccini, A. R.; Thomas, B. J. C.; Brusatin, G.; Colombo, P. Mechanical and Electrical Properties of Hot-Pressed Borosilicate Glass Matrix Composites Containing Multi-Wall Carbon Nanotubes. *J. Mater. Sci.* **2007**, *6*, 42.

(12) Müser, M. H.; Dapp, W.B.; Bugnicourt, R.; Sainsot, P.; Lesaffre, N.; Lubrecht, T.A.; Persson, B. N. J. et al., Meeting the Contact-Mechanics Challenge, *Tribol. Lett.* **2017**, *65*, 118.

(13) TriboNet. *TriboSolver Home Page*, 2023. https://tribology.eu/tribosolver/(accessed 09 20, 2023).
